# Nano and Microtechnologies for the Delivery of Oligonucleotides with Gene Silencing Properties 

**DOI:** 10.3390/molecules14082801

**Published:** 2009-07-29

**Authors:** Giuseppe De Rosa, Maria Immacolata La Rotonda

**Affiliations:** Dipartimento di Chimica Farmaceutica e Tossicologica, Facoltà di Farmacia, Università degli Studi di Napoli Federico II, Via D. Montesano 49, 80131 Naples, Italy; E-mail: larotond@unina.it (M.I.L.R.)

**Keywords:** oligonucleotide, siRNA, cationic liposomes, cationic polymers, PLGA microspheres

## Abstract

Oligonucleotides (ONs) are synthetic fragments of nucleic acid designed to modulate the expression of target proteins. DNA-based ONs (antisense, antigene, aptamer or decoy) and more recently a new class of RNA-based ONs, the small interfering RNAs (siRNAs), have gained great attention for the treatment of different disease states, such as viral infections, inflammation, diabetes, and cancer. However, the development of therapeutic strategies based on ONs is hampered by their low bioavailability, poor intracellular uptake and rapid degradation in biological fluids. The use of a non-viral carrier can be a powerful tool to overcome these drawbacks. Lipid or polymer-based nanotechnologies can improve biological stability and cellular uptake of ONs, with possibility of tissue and/or cellular targeting. The use of polymeric devices can also produce a prolonged release of the ON, thus reducing the need of frequent administrations. This review summarizes advantages and issues related to the main non-viral vectors used for ON delivery.

## Introduction

In the last decades, the discovery of the intracellular pathways responsible for a number of disease states has led to the identification of new therapeutic targets. Thus, many scientists have focused their attention on the development of new approaches to interfere with defective biochemical pathways, specifically by blocking the expression of a target protein. In this contest, a class of nucleic acid-based macromolecules, generically defined oligonucleotides (ONs), represents a powerful and promising tool. ONs are short fragments of DNA or RNA designed to specifically inhibit the expression of a target protein, by different mechanism of action. Antisense ON is a single stranded chain of DNA able to bind in a sequence-specific manner the target mRNA, consequently blocking its translation process [1]. An other approach consists on the use of a single stranded fragment of DNA that directly interacts with the target gene, thus hampering the transcription of the genetic information (antigene ON) [2]. A doubles stranded ON can also be designed to interact with a specific protein, such as a transcription factor, thus hampering its binding to the consensus sequence of target gene, blocking in turn the protein expression (decoy ON) [3]. Finally, the use of the SELEX technique allows to isolate fragments of DNA or RNA, also known as aptamers, able to bind a wide range of proteins of importance for therapy [4]. About 10 years ago, the discovery of the RNA interference (RNAi) has opened new perspectives for gene silencing. RNAi is a form of primitive immunity to protect the genome from invasion by exogenous nucleic acids [5,6,7]. RNAi relies on a multistep intracellular pathway in which a long double stranded RNA molecules (dsRNA) is processed by the endoribonuclease, named Dicer, in small interfering RNAs (siRNAs) [8]. These RNA fragments are then incorporated into a RNA-induced silencing complex (RISC) where the RNA duplex is unwound in a single stranded siRNA that allows recognition and cleavage of its complementary target mRNA [9,10]. The superiority of RNAi for gene silencing, compared with other approaches such as antisense, is due to the fact that after degradation of the target mRNA, RISC is recovered for further binding and cleavage cycles, resulting in higher reduction of the specific mRNA levels and hence into a lower expression of the correspondent gene. The possibility to use directly the siRNAs, rather than the long dsRNAs precursors, has been the key for the success of this approach.

In the last years, thousands of scientific papers focused on the therapeutic potential of ONs have been published. Moreover, different pharmaceutical and biotechnological companies have a number of silencing ONs in advanced phase of development for the treatment of different diseases such as hypercholesterolemia, cancer, inflammation, viral infections, neurodegenerative diseases. However, the great interest of the scientists on the therapeutic use of ONs seems in contrast with the poor success of these macromolecules on the market. In general, the development of a ON-based therapeutics is hampered by serious pharmacokinetic drawbacks. Indeed, the macromolecular and polyanionic nature of ON lead to a low bioavailability, thus requiring invasive administration routes. Moreover, ONs have a very short half-life in biological fluids due to the activity of nucleases [11,12]. Finally, once in contact with the target cell, ON can enter via clatrin-mediated endocytosis [13], probably by interaction with receptor of other anionic molecules such as heparin. However, the majority of the ON is degraded into the endolysosomes and only a negligible amount of ON can gain access to the cytoplasm and to the molecular targets. Thus, in the research of new therapies based on ONs, the main challenge is to overcome their disadvantageous pharmacokinetic profile. To this aim, chemical modifications of ONs, use of viral (in the case of the siRNA) and non-viral vectors have been largely investigated. In this review the more meaningful non-viral strategies for the delivery of ONs and for their use in therapy is described.

## Lipid-based Delivery Systems

### Cationic liposomes

Cationic liposomes are vesicles in which a cationic lipid-based membrane surrounds one or more aqueous cavities. Some of the most used cationic lipids are listed in Table 1. The addition of an ON solution to a liposome suspension induces vesicle aggregation and lipid mixing, due to the ionic interaction between the positive charges of the lipids and the negative charges of the nucleic acids. The resulting complexes are also named lipoplexes.

Cationic liposomes are certainly the most investigated non-viral delivery system for ONs. However, the variability of the transfection achievable by using cationic liposomes has incited the researchers to investigate all the variables affecting the efficiency of such a system. It was found that experimental conditions during lipoplex preparation significantly affect ON delivery. In particular, complexation obtained at ratios between negative charges of the nucleic acid and positive charges of the lipid (+/- ratios) far from the neutrality lead to small sized lipoplexes that generally results in a more efficient ON delivery [14,15,16]. Morphological characteristics of lipoplexes also depend on the type of cationic lipid, presence of neutral lipids, incubation medium, ratio between volumes of the nucleic acid solution and volume of the liposome suspension [14,15,17]. The ultrastructural analysis of lipoplexes prepared in different experimental conditions showed that the addition of ON to a cationic liposome suspension induced both aggregation and formation of a novel condensed lamellar phase [18]. This was explained with the effect of the polyanionic ON that can form bridges between the outermost membranes of neighboring particles, thus causing aggregation. The presence of neutral lipids affects lipoplex morphology, depending on the type of lipid. In particular, the presence of cholesterol in the formulation did not show a qualitative effect on the lipoplex morphology, but only an expansion of the interlamellar spacing [18]. On the contrary, the presence of a fusogenic lipid, such as dioleoylphosphatidylethanolamine (DOPE) (Table 1), in a mole fraction from 0.1 to 0.8, led to a structural transition from a lamellar (L_α_) phase to an inverted hexagonal (H_II_) phase with a region of coexistence in between [19]. The presence of salts when forming lipoplexes also favours the aggregation, especially in heterogeneous buffers such as media for cell culture [16,20]. Protein such as albumin lead to strong lipoplex aggregation, that should be the main reason for the *in vitro* ineffectiveness of lipoplexes in presence of serum [21]. After preparation, lipoplexes are not stable in liquid suspension for long-term storage and they aggregate. Therefore, it would be advantageous to develop dried formulations containing lipid/ON complexes that, upon rehydration, possess predictable physical characteristics and transfection efficiency comparable to fresh preparations. Lyophilization of siRNA/cationic liposomes complexes in ionic solutions lead to aggregation and lost of 65–75% of their functionality, although, inside the complex, the siRNA is unaffected and, hence, it is still active [22]. The use of lyoprotectants, such as glucose, can be of help to prevent loss in activity of siRNA-liposome, by preventing liposome fusion/aggregation [22].

**Table 1:** Lipids frequently used in the formulation of lipid-based non-viral vectors for ON delivery.

Chemical structure	Notes
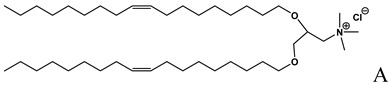	Cationic lipid used in the formulation of cationic liposomes [23,28,31,36]
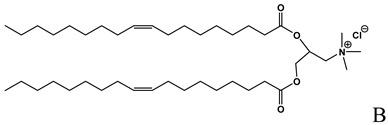	Cationic lipid used in the formulation of cationic liposomes and LPDs [15,16, 18,19,21,22,23,24,26,27,29,35,39,41,43,44,47,48,49,56],
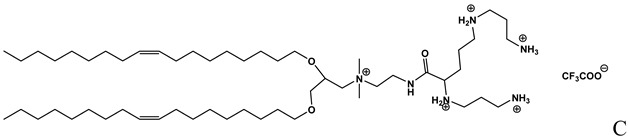	Cationic lipid used in the formulation of cationic liposomes [14]
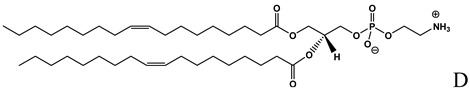	Helper lipid with fusogenic properties used in the formulation of cationic liposomes [14,15,16, 19,20,22,24,27,30,31,38,40,42,43,]
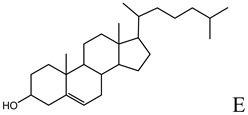	Helper lipid used in the formulation of cationic liposomes, LPDs, SNALPs and HVJ liposomes [16,18,20,27,33,36,47,48,49,50,51,52,53,55,56,57]
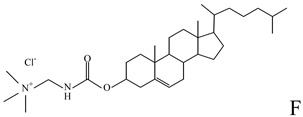	Cationic lipid used in the formulation of cationic liposomes and LPDs [38,55]
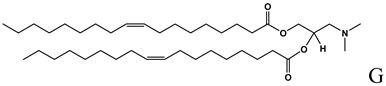	Ionizable lipid used in the formulation of cationic liposomes and SNALPs [24,50,51]
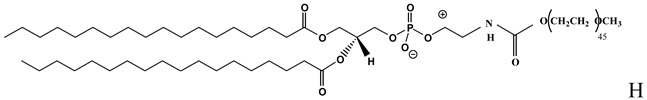	PEGylated lipid used in the formulation of LPDs [47,48,49]

A: N-[1-(2,3-dioleyloxy)propyl]-n,n,n-trimethylammonium chloride (DOTMA); B: 1,2-Dioleoyl-3-trimethylammonium-propane chloride salt (DOTAP); C: 2,3-dioleyloxy-N-[2(spermine-carboxamido)ethyl]-N,N-dimethyl-1-propanaminium trifluoroacetate (DOSPA); D: 1,2-dioleil-sn-glicero-3-fosfoetanolamine (DOPE); E: Cholesterol; F: 3ß-[N-(N',N'-dimethylaminoethane)-carbamoyl]cholesterol hydrochloride (DC-cholesterol); G: 1,2-dioleoyl-3-dimethylammonium-propane (DODAP); H: 1,2-diacyl-sn-glycero-3-phosphoethanolamine-N-[methoxy(polyethylene glycol)-2000] (DSPE-PEG2000).

The mechanism by which lipoplexes enter into cells and release the ON into the cytoplasm has been the object of many studies. The use of cationic liposomes markedly prevent ON degradation by nucleases in cultured cells and in human serum, thus improving the amount of intact ON delivered into the cytoplasm and nucleus [23,24]. It has been observed that once in contact with the cell membrane, the lipoplexes enter by clathrin-involved endocytosis [25,26]. Once into the endosome, the complex destabilize the endosomal membrane by a “flip-flop” mechanism of anionic lipids, mainly located on the cytoplasmic face of the membrane. Then, the anionic lipids diffuse into the complex neutralizing its positive charge and liberating the ON that diffuses into the cytoplasm [27]. After escape from the endosomes, ON accumulation into the nucleus has been observed [27,28,29]. Differently, siRNA have been found accumulate in perinuclear region of the cytoplasm, where the interaction with the RISC should “entrap” the nucleic acid; any fluorescence was found into the nucleus also after 24 h [30]. Chemical characteristics of the cationic lipid, its concentration and the incubation time with the cells, strongly affect the amount of ON uptaken into cells [28,31]. Moreover, an increased transfection efficiency can be achieved by inclusion of neutral lipids, also defined helper lipids (Table 1). Due to its fusogenic properties, DOPE is the most used helper lipid in the formulation of cationic liposomes. Once into the endosomes, DOPE segregates from the lipid mix, adopting an inverted exagonal phase (H_II_) that induces fusion of lipoplexes with the endosomal membrane and following entry of the nucleic acid into the cytoplasm [32]. Cholesterol has also been found to improve the transfection efficiency, but this effect has been attribute the a higher physical stability of complexes in cellular incubation medium [20,33].

It is well known that the use of cationic liposomes decreases cell viability. The cytotoxicity of lipoplexes have been attributed to the binding of cationic lipids to intracellular anionic lipids (e.g. cardiolipin), that can severely compromise the metabolic pathway of the cell [34].

Cationic liposomes have been successfully used for local delivery of ON. The intraarticular administration of a decoy ON against the transcription factor NF-κB, complexed with cationic liposomes prevented the recurrence of streptococcal cell wall-induced arthritis in treated joints [35]. The intraperitoneal administration of CpG ON complexed with cationic liposomes efficiently inhibited peritoneal dissemination in mice [36]. Cationic liposomes have also been used for mucosal applications. Palliser *et al.* showed that vaginal instillation of siRNA targeting herpes simplex virus 2 (HSV-2) protects mice from lethal infection [37]. A efficient uptake of siRNA/lipid complexes was shown in epithelial and lamina propria cells and silence gene expression in the mouse vagina and ectocervix for at least nine days was reported. Morever, the formulation was well tolerated, did not induce interferon-responsive genes or cause inflammation, and protected mice when administered before and/or after lethal HSV-2 challenge.

When systemically administered, the use of cationic liposomes alters the ON pharmacokinetic and distribution [38]. Upon intravenous administration, lipoplexes transiently accumulate into the pulmonary capillaries and gradually redistributed to the liver, mainly in the Kupffer cells [38,39,40]. This effect can be explained with lipoplex interaction with the serum components, especially albumin, which induces lipoplex aggregation [21] and mediates rapid uptake by the Kupffer cells of the liver [38]. In the light of these considerations, successful ON delivery to the liver can be obtained by systemic administration of lipoplexes. Sorensen *et al*. showed a successful silencing of the marker gene (GFP) in liver after intravenous injection of cationic liposome/siRNA complexes in mice [41]. On the other hand, the intravenous administration of siRNA complexed with cationic liposomes in an animal model of subcutaneous tumor did not result in a higher intratumoral levels of the nucleic acid, compared with siRNA administered as naked. This was explained with the rapid clearance of the complexes from the bloodstream. However, carrier-induced changes in the siRNA intratumoral distribution were evidenced and this could justify the higher activity of the ON when delivered by lipoplexes [39]. The use of targeting moieties, can significantly improve ON delivery to the target tissues. Sato *et al.* reported that siRNA could be efficiently delivered to liver parenchymal cell by using galactosylated cationic liposomes [42]. Targeted delivery of siRNA to primary and metastatic tumor cells was demonstrated with immunoliposomes bearing an anti-transferrin receptor single-chain antibody fragment after intravenous administration [43].

Despite of same interesting results, the potential toxicity in the use of siRNA/cationic liposomes suggests caution in their *in vivo* use by systemic administration. This was underlined by Mao *et al*. who found that, although intravenous administration of siRNA alone is essentially inert, injection of cationic lipid/siRNA complexes resulted in a potent induction of both type I and type II IFN response. Furthermore, intravenous administration of cationic liposome/siRNA complexes led to activation of STAT1 [44]. The toxic effect of lipoplexes have also been evidenced in two phase I clinical trials, suggesting that a further optimization of the formulation characteristics is still required [45,46]. Thus, the necessity to get out of the *in vivo* toxicity issues, together with the aspecific distribution and the variability of the performance, remain the main obstacles to move cationic liposomes/ON complexes from the animal model to the clinical practice.

### Other lipid-based delivery systems

Cationic liposomes have also been used to cover protamine-DNA-ON complexes. The resulting liposome-polycation-DNA (LPD) nanoparticles can then further be covered with DSPE-PEG in order to increase physical stability in serum [47]. The binding of anisamide, a small molecule that targets the sigma receptor overexpressing cells, e.g., human lung cancer cells, significantly improve LPD localization in lung cancer [48]. LPD nanoparticles efficiently penetrated the lung metastasis, with a 70-80% of gene silencing, while uptake into the liver was not reported [49]. Although few *in vivo* data are available in the literature, the easy preparation together with the low immunotoxicity [49] make this system very interesting for further studies.

A significant improvement for the delivery of ON was the development of the stabilized antisense lipid particles (SALPs), today known as stable nucleic acid lipid particles (SNALPs). Semple *et al*. described the encapsulation of ON in liposomes with an ionisable lipid (Table 1), by which it is possible to achieve high ON encapsulation efficiency due to the presence of positively charged lipid and neutralization of the liposome surface after preparation [50]. Moreover, the stability of the suspension in presence of serum as well as a long *in vivo* half-life are assured by PEG moieties on the liposome surface. This system has showed promising results for the delivery of an anticancer antisense ON [51] and for antiviral siRNA [52] in mouse. Moreover, SNALPs have been successfully used to systemically deliver a siRNA anti-apolipoprotein B in non-human primates (cynomolgus monkey), leading to a dose-dependent silencing effect > 90% after 48 h [53]. The biological effect was immediate, as early 24 h after the treatment, and lasting for 11 days. The biological effects where the reduction of the blood levels of ApoB-100 protein, total cholesterol and LDL. In the case of cholesterol, the levels of reduction were exceeded the levels obtained with currently approved cholesterol-lowering drugs [53]. The silencing effect was observed only in the liver, thus suggesting the great potential of SNALP for therapeutic applications of siRNA for liver-based diseases. Interestingly, no complement activation, delayed coagulation, pro-inflammatory cytokines production or changes in haematological parameters were found upon systemic use of SNALP in monkeys [53]. These exciting results have led to a rapid movement of this delivery system from the lab to the clinic and on the July 5, 2009, Tekmira Pharmaceuticals Corporation announced that it has initiated a Phase 1 human clinical trial for siRNA anti-ApoB delivered by SNALP. The results of this study are expected in early 2010.

A different strategy to improve the ON delivery by cationic liposomes consists of the insertion of fusion proteins into liposome membrane. In particular, hemagglutinating virus of Japan (HVJ), also known as Sendai virus, is able to fuse with cell membrane and with liposomes [54]. ON-loaded liposomes are fused with UV-inactivated HVJ to form HVJ liposomes [55]. Compared with classic lipoplexes, HVJ cationic liposomes enhance the delivery of ON into cultured cells with preservation of the ON integrity. This effect has been attributed to the fusion of the HVJ-cationic liposomes with the cell membrane that avoids ON degradation into the endolysosomes [55]. With the same strategy, HVJ-liposomes based on cationic [56] neutral lipid were developed to efficiently deliver ON *in vivo* [57,58]. However, for a larger use of these carriers, the risk of immunogenicity have to be seriously taken into account.

## Polymer-based Delivery Systems

### Poly(ethyleneimine)

Polyethylemimine (PEI) is a polymer characterised by a high density of positive charges, available in different molecular weight and in linear or branched form (Table 2) [59]. PEI is able to interact with nucleic acids to form complexes able to efficiently enter into cells [60]. The physical characteristics of ON/PEI complexes depend on different experimental variables during complex formation. Firstly, the medium in which the complexes are prepared, the type of PEI (molecular weight and linear or branched form), the ON concentration and the N/P ratio have to be carefully selected in order to obtain nanosized ON/PEI complexes [61,62]. Reduced complex aggregation can also be obtained by using low molecular weight PEI or PEI-PEG conjugates. Indeed, the presence of PEG increases the water solubility of complexes, thus improving their physical stability [63]. The use of high molecular weight PEG further contributes to reduce complex size, due to a higher steric shielding effect of PEG that abolishes aggregation [64,65]. The level of PEG grafting as well as the PEI molecular weight can affect the stability of ON/PEI-PEG complexes [64]. From a morphological point of view, the ON/PEI complexes appear as spheroid nanoparticles with a core-shell structure in which the complex completely cover free nucleic acid [61]. Interaction with PEI prevents ON degradation by nucleases, but this effect can be slightly different depending on the PEI molecular weight and conjugation with PEG [65,66,67]. Lyophilization does not alter morphology and transfection efficiency of ON complexes with PEI and PEI-PEG conjugates [63,68], allowing the long term storage of complexes. The high transfection efficiency of PEI has been attributed to its buffering effect, also known as “proton sponge effect”. Once into the endolysosomes, PEI/ON complexes, due to the high density of the free amino groups, accept protons pumped into the vesicles thus inducing a further proton entry together with an influx of Cl^-^.

**Table 2:** Polymers frequently used in the formulation of non-viral vectors for ON delivery.

Chemical structure and name	Notes
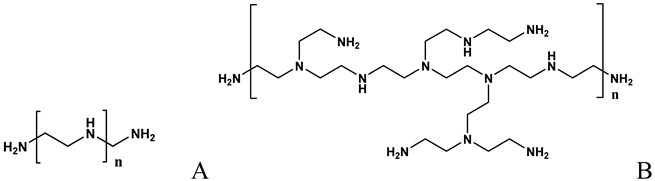	Formation of complexes (polyplexes) by ionic interaction with ON [60,61,62,64,65,67,71,73,74,75,79]
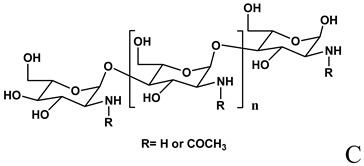	Entrapment of ON through different mechanisms, including ionic crosslinking, desolvation, or ionic complexation[76,84,85,86,87,88,89,90,91,92,93,94]
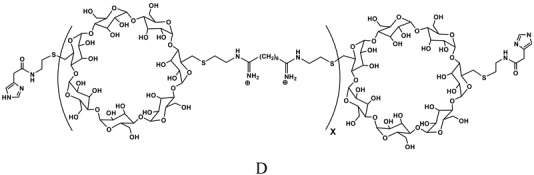	Formation of complexes with ON by ionic interaction [73,98,99,100,101,102,103,104,105,106,109,110]
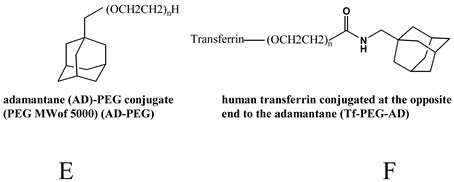	Hydrophobic interaction with CDP [73,104,106,107,108,109,110]
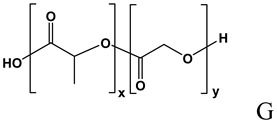	Physical entrapment of ON [113,114,115,116,117,118,119,120,121,122,123,124,125,126,127,128,129,130,131,132]

A: Linear polyethylenimine ; B: branched polyethylenimine; C: chitosan; D: cyclodextrin containing polymer (CDP); E: adamantane conjugate (AD-PEG); F: human transferrin conjugated to the adamantane (Tf-PEG-AD); G: poly(lactide-co-glycolide).

The increased lysosomal osmolarity leads to the water rushing to relieve the gradient, resulting in lysosomal swelling and bursting [69]. The presence of PEI could raise endolysosomal pH, which, in turn, could alter protein folding within the endolysosome and perhaps inactivate degradative enzymes. This should avoid the enzymatic degradation of the nucleic acid before endolysosomal bursting [69]. PEGylation only barely affects buffer capacity of PEI [64]. After complex escape from endolysosomes, nuclear co-localization of nucleic acid and PEI has been found in the case of plasmid DNA. In particular, it has been hypothesized that a nuclear entry of the complexes is mediated by interaction with intracellular anionic lipids [70]. Thus, it is reasonable to hypothesize a similar mechanism in the case of short ON, i.e. antisense ON, with a nuclear target. However, in the case of ON/PEI-PEG complexes, only the nucleic acid has been found to diffuse into the nucleus [71]. Moreover, in the case of siRNA, the complex dissociation in the cytoplasm should arrive, thus leading the interaction of the nucleic acid with the cytoplasmic RISC. Further studies are needed to better clarify the intracellular fate of PEI or PEI-PEG, after ON delivery.

The efficiency of PEI as intracellular carrier for ONs has been shown on different cell lines. The transfection efficiency have been found to depend on complex characteristics such as size, zeta potential and stability of ON/PEI (or ON/PEI-PEG) complexes [61,62,65,71,72,73,74]. However, PEI also reduce cell viability in a concentration dependent manner [61,62]. Different hypotheses have been formulated to justify PEI cytotoxicity, such as a permeabilization effect on cell membrane and lysosomal destruction [69].

Fisher and collegues [75] investigated the ON and PEI pharmacokinetics and biodistribution, upon intravenous injection in mice. When compared with the administration of free ON, PEI complexes decreased the clearance of the ON from the circulation. Moreover, a different ON pharmacokinetic profile was found depending on the PEI molecular weight and conjugation with PEG. This was attributed to a different binding to plasma cells that was observed for the different form of PEI [76]. Moreover, with regard to the pattern of organ accumulation, the administration of ON/PEI-PEG complexes resulted in an ON distribution profile in the body very similar to that of free ON. In particular, after 15 minutes from the injections, the higher ON concentrations were found in liver and kidney. On the other hand, the use of PEI, independently of the molecular weight, leads to reduced (> than 50%) ON accumulation into the kidney, and to a much higher accumulation into the liver and spleen. In the case of 25kDa PEI, relatively high ON concentrations in heart, lung and fat tissue were also found [75]. These findings can be explained with the weaker binding between complex components in the case of PEG-PEI, as demonstrated by Glodde *et al*. [64]. Differently, in the case of unmodified PEI, a rapid complex opsonization and subsequent clearance by phagocytic macrophages of reticuloendothelial system seems to occur. PEI has been successfully used to deliver ON by systemic administration in different animal model [77,78]. Interestingly, the full bioactivity of ON delivered by PEI was found at non-toxic concentrations [78]. Moreover, in the case of low molecular weight PEI, the systemic use did not induce an inflammatory response [79]. The transfection efficiency of PEI or PEI-PEG conjugates can be further improved by conjugation with ligands able to bind specific receptors on the target cells, thus stimulating receptor-mediated endocytosis. The delivery of siRNA with PEI conjugated with hyaluronic acid, for example, significantly improved gene silencing in cells overexpressing the receptors for hyaluronic acid [80]. Surface modification of polyplexes with cell penetrating peptide TAT (trans-activator of transcription) further improved the exon-skipping ON transfection efficiency, significantly improving dystrophin expression in an animal model of Duchenne muscular dystrophy [81]. However, the use of a PEI functionalized with targeting moieties did not result in a higher ON localization into tumor, compared with free ON, but rather in a different ON distribution into the tumor [39]. PEI is certainly one of the most investigated non-viral carrier. However, despite the good performance demostrated on different animal model, at our knowledge, any clinical study for ON delivery have been carried out. This could be due the high variability of polyplex characteristics that can affect the ON delivery. On the other hand the use of PEGylated PEI can be of help to control morphological characteristics, although it forms less stable complexes, resulting in a lower transfection effiency. Morever, toxicity issues due to PEI accumulation after a long term use should be carefully evaluated.

### Chitosan

Chitosan (Table 2), a linear β-(1,4)-2-amino-2-deoxy-D-glucose homopolymer, is a deacetylated form of chitin, an abundant polysaccharide present in crustacean shells. In the last two decades this polymer has received great attention as a material for biomedical and drug delivery applications. This polymer is biodegradable, biocompatible and not much toxic [82,83]. Then, from a technological point of view, chitosan is hydrosoluble and positively charged. These properties enable it to interact with negatively charged macromolecules, such as nucleic acids, in an aqueous environment.

Chitosan has been shown to form colloidal particles and entrap macromolecules through a number of mechanisms, including ionic crosslinking, desolvation, or ionic complexation. When prepared by complexation, nanoparticle characteristics depend on the molecular weight, degree of deacetylation and concentration of the polymer [76,84,85]. In the case of preparation by crosslinking, also the ratio between chitosan and cross-linker, pH and loading method affect nanoparticle properties [84,86]. Once complexed with chitosan, ON is efficiently protected from the enzymatic degradation independently from the N/P ratio [87].

Chitosan molecular weight, degree of deacetylation, N/P ratio as well as the preparation method of the nanoparticles strongly affect trasfection efficiency [84,85]. For example, chitosan prepared by ionic gelation using sodium tripolyphosphate were more efficient to deliver siRNA compared to chitosan-siRNA complexes, possibly due to their high binding capacity and loading efficiency [84]. Long term storage of chitosan/siRNA complexes without loss of transfection efficiency can be achieved by complex lyophilization in presence of sucrose as lyprotectant [88]. The limited cytotoxicity of chitosan can be further reduced by using a low molecular weight chitosan [89]. Galactosylation of low molecular weight chitosan can result in a further reduction of cytotoxicity, without loss of trasfection efficiency [89].

Chitosan significantly changed ON pharmacokinetics and biodistribution after intravenous administration, with a higher ON half-life [90] and higher ON accumulation within the liver [91]. The use of galactosylated-chitosan can be useful to achieve a preferential targeting toward the Kupffer cells rather than into liver parenchymal cells [91]. With this kind of polymer, it was showed an enhanced delivery of an antisense ON targeting the TNF-α into Kupffer cells in the treatment of fulminant hepatitis. In particular, the intravenous injection of ON/galactosylated-chitosan nanoparticles, compared with the naked ON, led to a notably reduction of the dose administrated in animals and to a prolonged effectiveness [92].

The problem of nanoparticle instability and clearance associated with the intravenous administration can be overcame by intraperitoneal injection of chitosan/ON nanoparticles [93]. By this approach, chitosan/siRNA nanoparticles entered peritoneal macrophages, downregulated TNF-α-induced inflammatory responses and arrested joint swelling in a model of collagen-induced arthritis [93].

Chitosan have also been used for topical delivery of ON. A topical chitosan/siRNA nanoparticle formulation has been used for transdermal delivery to the lung and to reduce airway hyperresponsiveness in mice [94]. Anti-inflammatory effects including reduced airway hyperresponsiveness, eosinophilia, lung histopathology and reduction in pro-inflammatory IL-4 and IL-5 in lung homogenates, were reported in mouse model for asthma, after topical application of the chitosan formulation compared to scrambled control. Moreover, ON delivery through the nasal mucosa have been reported. Intranasal delivery of chitosan nanoparticles containing a siRNA (phosphodiester and chemically modified small interfering locked nucleic acid or siLNA) targeting enhanced-green-fluorescent-protein (EGFP) in lung bronchoepithelium efficiently reduces the EGFP protein expression similarly to naked siRNA administered intravenously. In the same experimental model, intranasally instilled naked siRNA did not cause a knockdown of the target protein [76,95]. In a murine model of allergic rhinitis, the IL-5 and IgE levels closely related to the allergic inflammation were significantly reduced after the intranasal administration of an anti-IL-5 ON complexed with chitosan [87].

Among the non-viral vectors, chitosan has certainly demonstrated the most interesting performance for transdermal delivery of ON. Otherwise, the number of biological effect reported [83] for this polymer have to be seriously taken into account when using chitosan by systemic administration.

### Cyclodextrin-containing polymers

Cyclodextrins (CDs) are cyclic polysaccharides largely used in pharmaceutical formulations, and as such, with a long-term biocompatibility well known in humans [96,97]. Because of their low toxicity, the lack of immune stimulation and the absence of enzyme degradation in humans, CDs have been chosen as one of the building blocks for a new polymer for delivery of nucleic acids (Table 2). In particular, a new class of polymers, in which the CD is part of the backbone of a linear, water-soluble copolymer, has been developed. The structure−functional behaviour of these CD-containing polymers (CDPs) has been the object of numerous studies [98,99,100,101,102,103]. Different CDPs have been synthesized in order to obtain low toxicity, minimal complement activation and to permit renal clearance in animals and humans. In particular, the presence of the CD was important to confer the highest water solubility and the lowest toxicity. Beta-CD was chosen because it provided much better properties for large-scale production of CDP over both alpha and gamma-CD. Moreover, the distance between the CD and the charge center and the type of charge centers were investigated in order to obtain the lowest toxicity and a better delivery efficacy [104]. The addition of the imidazole termini did provide buffering of endocytic vesicles in living cells [105]. Steric stabilization was obtained by non covalent interaction between CDs of the copolymer and adamantine (AD) conjugated with PEG (Table 2). The attachment of ligands, i.e. galactose and transferrin (Tf), to the free extremity of the PEG-AD (Table 2) allowed to achieve nanoparticles for cell targeting [106,107,108]. The nanoparticles based on CDP and complexing the nucleic acid can be prepared by placing in one vial the mix of CDP, PEG-AD and ligand-PEG-AD, and the nucleic acid in a second vial. When the two solutions (same volumes) are mixed, the nanoparticles self-assemble [106]. A typical formulation involves a 3:1 charge ratio of polymer positive charge to nucleic acid negative charge, assuring all the nucleic acid is contained within the carriers. The nanoparticles have approximately a 1:1 charge ratio, so the excess of CDP is in solution. Also, since a 1:1 mol ratio between the cyclodextrin and the AD from the total PEG-AD and ligand-PEG-AD is used, there is an excess of PEG-AD and PEG-AD-ligand in solution [106]. Nanoparticle diameters of CDP formulations with siRNA increase with nucleic acid concentration (exceeding 100 nm) when AD-PEG is not included at the time of formulation [106]. However, when AD-PEG is included, the nanoparticle diameter remains constant, independent of nucleic acid concentration, at 60−80 nm [106]. Davis reported that siRNA and CPD were able to assemble in the circulation of the mouse, thus excluding the disassembling of the siRNA/CDP complexes in the circulation [104].

The *in vivo* efficiency of the CDP-based delivery system complexing siRNA targeting the breakpoint of the EWS-FLI1 fusion gene was showed in a disseminated murine model of Ewing’s sarcoma [104]. Interestingly, only the formulation containing a targeting moiety (i.e. transferrin) showed antitumor effect. Following studies evidenced unchanged biodistribution of targeted and non-targeted nanoparticles [109]. Thus, the primary effect of the Tf targeting ligand is not to improve the amount of carrier localized in the tumor tissue, but to enhance, once into the tumor, the intracellular uptake [109]. *In vivo* experiments on a non-human primate (cynomolgus monkeys) have been also carried out [109,110]. In this study, a siRNA targeting the M2 subunit of ribonucleotide reductase (RMM2) was used. At different doses the nanoparticles were well tolerated and no complement activation and liver toxicities were observed. However, elevated levels of blood urea nitrogen and creatinine were observed, indicating mild and reversible kidney toxicity, associated to high IL-6 levels. Low titer, non-clearing (no changes in PK) antibodies to the human Tf on the nanoparticles were found. It is worthy of note that the antibodies were not against the PEGylated nanoparticles. This is important, as clearing antibody generation in humans has been observed with PEGylated liposomes. In total, the multiple, systemic doses of the targeted nanoparticles containing the unmodified siRNA were safely administered to non-human primates [109,110]. At the moment, a CDP-based formulation containing an unmodified siRNA against RRM2, and employing Tf as targeting agent is under phase I of clinical trial. The trial is a safety study treating adults with solid tumors that are refractory to standard-of-care therapies.

### Poly(lactide-co-glycolide) nano and microspheres

Poly(lactide) (PLA) and its copolymers with glycolic acid, poly(lactide-co-glycolide) (PLGA) (Table 2), are biodegradable polyesters extensively used as drug delivery platforms [111]. This family of polymers and copolymers is available on the market with different molecular weights, lactide to glycolide ratios and hydrophilicity (i.e. capped and uncapped end-groups), offering the possibility of choosing among very different biodegradation rates [112]. ON loading into PLA-based polymeric devices offers advantages such as a sustained release and an efficient protection against serum nucleases, without loss of the hybridization capability [113]. The possibility to formulate biodegradable polymers under the form of microspheres allows to directly inject the delivery system into the administration site without the need of a surgical implantation. Moreover, the use of biodegradable polymers avoids the need to remove the device after the complete drug release. The increased serum stability of antisense ON, when encapsulated into PLGA microspheres, have been clearly showed [114,115]. Different techniques have been used to encapsulate ON into microspheres; among them, the water-in-oil-in-water emulsion/solvent evaporation is the more frequently used [115,116,117,118]. With this technique, a number of experimental variables need to be carefully taken into account in order to achieve the particles with the expected technological properties. In particular, type of polymer, ON length and chemistry, ON loading, microspheres size, and the use of additives in the internal or in the external aqueous phase, strongly affect encapsulation efficiency and release properties [114,115,116,118,119,120,116,118]. High drug loading is generally associated with a larger size [121], but is independent of ON length [115]. ON *in vitro* release profile is generally very rapid in the case of small particles (mean diameter of about 1-2 μm), while an increase in size (mean diameter of about 10-20 μm) can lead to a significant slowing of ON release rate [121].

Biodegradable microspheres have been designed with different aims, depending on the microsphere characteristics. Thus, nanospheres or small microspheres (mean diameter lower than 4-5 μm), have been designed to be uptaken by cells, thus improving the intracellular concentration of ON [121,122,123,124,125,126]. Particle uptake occurs by an saturable and temperature dependent phagocytosis [121]. In this case, particles changed ON cellular distribution, although the presence of a diffuse or punctuate localization in the cytosol or the nuclear localization depends on the particle release properties [121,126]. In particular, after the particle uptake from cells, a lower ON release rate was considered to favour a higher ON accumulation into the nucleus [126]. Contrarily to small particles, large microsphere (diameter higher than 5 μm) are unable to be taken up by cells. These microsphere are mainly designed to provide a long term delivery of the ON, thus reducing the need of frequent administrations. In this case, the release rate can play an important role in cellular uptake. Our group demonstrated that, at a fixed concentration, ON cell uptake is enhanced by slow release, compared with ON administered in naked form. In particular, a higher ON accumulation into the cytoplasm was found in the case of single or double stranded ON [114,116,117]. The contemporaneous release of ON with the cationic polymer PEI resulted in an further increase of the ON entry into the cytosol, with ON accumulation into the nucleus. The optimization of the technological characteristics, such as reduction of the microsphere surface porosity, can allow to modulate ON release profile from microspheres, thus leading to a further increase of ON intracellular uptake [114]. Cell culture studies showed that the ON released from microspheres is still active; moreover, when slowly released from microspheres, the ON had an effect about 80 fold higher than that obtained with naked ON [117]. These findings has been explained by the fact that ON entries into cells prevalently by a endocytosis process [127]. Thus, at high ON concentrations, the endocytosis can be saturated and a higher dose fraction of ON can be degraded by exonucleases. When encapsulated into PLGA microspheres, ONs are efficiently protected from the enzymatic activity; in this case, only a little percentage of the administered dose is released from microspheres and pinocytotic saturation is not expected to occur with a higher percentage of ODN that can enter into cells.

*In vivo*, PLGA microspheres significantly increased the ON persistence at the administration site, although biodistribution into liver, kidney and spleen was not affected [128]. The efficacy of the ON slowly released from microspheres have been showed in different *in vivo* experimental models. In particular, a high efficacy of the ON and a long term effect have been demonstrated. In animal models of melanoma, the subcutaneous administration of microspheres encapsulating the antisense ON targeting the proto-oncogene c-myc, resulted in a reduced tumor growth, decreased number of metastases, reduced c-myc expression, and increased survival, compared the ON in naked form [129]. In the same study, a decreased metastatic potential and an increased survival were also observed in the leukemia model. In a balloon-injured rat restenosis model, PLGA nanoparticles containing an antisense ON against platelet-derived growth factor beta-receptor showed an antirestenotic effect after 14 days from the treatment [125]. PLGA nanoparticles containing CpG ON (used as immunostimulant) resulted in antigen specific T cell proliferation in Balb/c mice receiving immunogenic tetanus toxoid. The effect was obtained with naked CpG ON, but at 2-orders of magnitude higher dose [130]. Recently, our group reported that in an animal model of chronic inflammation, the subcutaneous injection of PLGA microspheres releasing decoy ON against the transcription factor NF-κB, resulted in inhibition of inflammation up to 15 days (time at which the experiment was concluded). In the same experimental model, naked ON, at the same administered dose, showed a similar effect only from 1 up to 5 days [131]. The so-called “Trojan delivery system” consisting of PLGA microparticles releasing ON/PEI complexes was tested *in vivo*. In an animal model of glaucoma, rabbit treatment with PLGA microspheres releasing an anti Transforming growth factor β2 antisense ON prolonged bleb survival for 28 days (50% of the treated eyes) following trabeculectomy compared to control. This effect was significantly higher in the case of PLGA microspheres releasing ON/PEI complexes with a bleb survival of 42 days on the 100% of the treated eyes [132]. Sirsi *et al*. found that PLGA releasing an exon-skipping ON complexed with PEI-PEG resulted in a significant increase of dystrophin-positive fibers; however, the authors did not find a significant advantage compared to ON/PEI-PEG complexes, underlining the usefulness of PLGA system only on long time range [120].

## Conclusions

The first ON-based therapeutic product reaching the market was Vitravene^®^ (containing Formivirsen) an antisense ON indicated for the local treatment of cytomegalovirus retinitis in patients with AIDS. This product received the marketing authorization in 1998 in USA and in 1999 in the European Union. However, the marketing authorization holder decided in 2002 to withdraw the product from the European Union. The decision was due to commercial reasons (the demand was only about 100 patients/year) and not to any safety concerns. The commercial failure of the first marketed ON-based drug did not discourage the researchers and, in the last years, a growing number of new ONs, especially siRNAs, have been proposed for therapeutics applications. Companies such as Isis Pharmaceuticals and Alnylam have different ONs in advanced phase clinical trials. However, the delivery of ON into the target cells or organs remains the major drawback to the therapeutic application of these product. The use of non-viral vectors remains the most attractive approach due to the easy preparation, low immunogenicity and safety. The growing number of *in vivo* experiments are confirming the efficacy of some non-viral carriers, and exciting results have been also obtained on non human primates, especially in terms of safety and side effects. Further efforts are still required to improve drug targeting and long term safety studies are still needed. The first clinical trials started in 2008 with targeted nanoparticles developed by Calando Pharmaceuticals, and the recently started clinical study on SNALP announced by Tekmira Pharmaceuticals Corporation suggests that the use of new products based on a silencing strategies for therapeutic purposes will occur in the not too distant future.
